# Competitive Rationales: Beneath the Surface of Competitive Behavior

**DOI:** 10.1177/01492063211040555

**Published:** 2021-09-04

**Authors:** Goce Andrevski, Danny Miller, Isabelle Le Breton-Miller, Walter Ferrier

**Affiliations:** Queen’s University; HEC Montreal; University of Kentucky

**Keywords:** competitive rationales, competitive intent, competitive dynamics, tactical actions, strategic forbearance, managerial cognition

## Abstract

Competitive dynamics research has focused on studying whether rivals are able and likely to carry out competitive actions, typically by examining indirect reasons such as characteristics of the actions themselves, the firms involved, or the competitive context. We explore why rivals initiate a specific competitive action at a particular time and situation. Drawing from the philosophy of action literature, we introduce the concept of competitive rationales to examine the primary reasons that cause tactical actions. Given the rapid exchanges characterizing tactical competitive dynamics, we conducted an inductive, multicase study to explore the reasons behind over 800 discrete tactical decisions carried out by 9 professional basketball coaches during 15 basketball games. To garner insight, we develop a conceptual framework revealing their types and scope. Even during intense head-to-head rivalry, most rationales were not rivalrous but were instead organizational—to optimize resource use, strategic consistency, and reputation—or social—to manage relationships. Moreover, the three main types of rationales varied in scope, extending beyond immediate competitive situations and rivals to address longer term, strategic outcomes, and assorted stakeholders. Thus, our analysis reveals these rationales to be complex and potentially difficult for rivals to decipher. It also recasts each component of the dominant awareness-motivation-capability (AMC) model of rivalry, suggesting that awareness is challenged by subtle rationales, motivation drives not only action but also forbearance, and capability is both a requirement and product of action.

## Introduction

Competitive action is the central construct and a key distinguishing feature of competitive dynamics research (Chen & Miller, 2012, 2015). Defined as “a specific and detectable competitive move, such as a price cut or a new product introduction initiated by a firm, to defend or improve its relative competitive position” (Smith, Grimm, Gannon, & Chen, 1991: 61), competitive actions are essential components of competitive attacks and responses (Chen & Miller, 1994), competitive aggressiveness (Andrevski & Ferrier, 2019; Ferrier & Lee, 2002), competitive complexity (Connelly, Tihanyi, Ketchen, Carnes, & Ferrier, 2017), and inertia (Miller & Chen, 1996).

Unfortunately, the *primary reasons* why managers carry out specific competitive actions and responses remain underexplored. Competitive dynamics research has examined various factors that drive competitive actions: superior capabilities (e.g., Andrevski, Brass, & Ferrier, 2016; Carnes, Xu, Sirmon, & Karadag, 2019; Gnyawali & Madhavan, 2001; Ndofor, Sirmon, & He, 2011; Smith et al., 1991), market centrality and multimarket contact (e.g., Chen & Miller, 1994; Livengood & Reger, 2010; McGrath, Chen, & MacMillan, 1998; Yu & Cannella, 2013), subjective perceptions of rivalry and tension (e.g., Chen, Su, &Tsai, 2007; Kilduff, Elfenbein, & Staw, 2010), and managerial cognitive dispositions (e.g., Hambrick, Cho, & Chen, 1996; Nadkarni & Barr, 2008; Nadkarni & Chen, 2014). However, those factors are not the primary reasons for a specific action. They describe what enables, stimulates, and hinders the motivation to carry out *any* competitive action. For example, competitive tension or superior capability can tempt action but cannot explain why a firm initiates a specific action at a particular time or situation, nor what a firm is attempting to achieve with that action.

Drawing on the philosophy of action literature, we introduce the construct of *competitive rationale* in studying the *primary* reasons that directly cause a specific competitive action or inaction. We apply the causal theory of action to provide theoretical underpinnings of competitive rationales (Davis, 2013; Davidson, 1963; O’Connor, 2013). This theory “can explain a person’s actions by citing the reasons for which (s)he did them” (Davis, 2013: 35). Primary reasons are an agent’s own reasons for performing an action. They describe what an agent wants to achieve and why it carries out that *particular* action, which is a prerequisite for predicting competitive behavior, a central goal of competitive dynamics research (Chen & Miller, 2012). In seeking these reasons or competitive rationales, we hope to reveal both their principal types and scope domains.

Most research has studied drivers of strategic rather than tactical actions, which, by comparison, require less time, expertise, and resources to develop and implement. Yet firms compete predominantly via tactical actions, often by middle-management—a reality evident in empirical studies of competitive aggressiveness, inertia, and simplicity (Ferrier, 2001; Ferrier & Lee, 2002; Ferrier, Smith, & Grimm, 1999; Miller & Chen, 1994, 1996) and by work showing how tactics can overwhelm rivals and neutralize attacks (Andrevski & Ferrier, 2019). Because tactical behavior embodies, implements, and adapts strategy (Chen & Miller, 2015), it tends to reflect “strategic” purpose (Van den Steen, 2018).

Our study explores competitive rationales—*primary reasons*—that directly cause tactical actions. It asks: *What types of rationales drive tactical actions, and what is their scope in competitive rivalry?* Given the rapid exchanges characterizing tactical competitive dynamics (Chen & MacMillan, 1992), we conducted an inductive, multicase study to examine the reasoning behind over 800 discrete tactical decisions carried out by 9 professional basketball coaches during 15 basketball games. We interviewed coaches and asked them to elaborate on their rationales for each considered action. The context enabled us to concentrate on tactical actions, as when a game is in progress, coaches have limited time and resources to execute. In addition, owing to its simplicity vis-à-vis business situations, our setting is an exacting one for discovering the strategic role of tactics. “Studying a case that is likely to display the lowest incidence or intensity of some feature in a population, we can assume that the rest of the population is above the level identified” (Gomm, Hammersley, & Foster, 2000: 108). Indeed, compared to most midlevel tactical business managers, basketball coaches have less time, resources, or opportunity to devise and execute tactics during a game. Thus, if tactical rationales are shown to be nuanced and diverse where tactics are executed with limited resources in seconds, they are likely to be even more so in business contexts (Grimm & Smith, 1997).

Our conceptual framework for competitive rationales consists of two dimensions: rationale type and rationale scope. First, competitive rationales are not solely rivalrous but more often organizational and social. In fact, rivalrous rationales—those primarily oriented to outmaneuver competitors—represented just one third of the total. Surprisingly, even in intense head-to-head tactical competition, most rationales were organizational, intended to orchestrate resources, learn through experience, and improve strategic coherence and execution. Other types of rationales were social, focusing on managing relationships with stakeholders. Second, the scope of rationales varies across three distinct domains: the immediate situation with rivals, those relating to longer term outcomes, and those involving stakeholders other than rivals. We found that 67% of rationales were intended not only to develop, preserve, and optimize resources but also to achieve temporally extended objectives, adjust strategy, and manage stakeholder relationships.

Our findings suggest important modifications to the dominant AMC model of competitive dynamics. We find that the complexity and inscrutability of rationales conceal rival intentions, thereby challenging and recasting the *awareness* component of the AMC model. Understanding the type and scope of each rationale can help interpret actions. Regarding *motivation*, we find rationales to underlie the motivation not only to act but also to strategically *forbear*, surfacing motivations pertaining to more distal time intervals, parties other than current rivals, and strategies more than unitary moves. Finally, whereas previous research has *capability* driving actions, we found the opposite: Actions often aim to build capabilities by dynamically managing resources for future advantage. In short, tactical actions constitute subtle and multifaceted vehicles for achieving long-term, strategic objectives, thereby broadening current understandings.

In the pages that follow, we provide a theoretical foundation of the concept of competitive rationales before describing our guiding research questions, methodology, conceptual framework and findings, and the implications of our study for the AMC model and more generally. Table 1 highlights the distinctive orientation and findings of our study and compares it to previous work in competitive dynamics.

**Table 1:** Distinctive Aspects of Our Study and Principal Findings

Distinctive Aspects	Typical CD Studies	Our Study of Rationales
Research focus	Strategic decisions	Tactical decisions
	Indirect reasons and goals that incentivize managers to act	Immediate reasons about “why” a coach initiates a specific action in a given time and situation
Research method	Rationales inferred from observable attributes	Rationales directly accessed from coaches’ statements
	Quantitative analysis	Qualitative analysis
	Deduction	Induction
Principal Findings	Typical CD Studies	Our Study of Rationales
Reasons for action	Indirect reasons that can affect *any* action	*Primary* reasons that directly cause *a specific* action or inaction
Types of rationales	Rivalrous rationales	Rivalrous, organizational, and social rationales
Scope of rationales	A single target domain: immediate rival, actions, outcomes	Three target domains: immediate situation, long-term strategic outcomes, and other implicated parties beyond a direct rival
Nature of rationales	Simple, parsimonious indirect factors that stimulate strategic actions (e.g., attack irreversibility; firm size, market commonality, cognitive dispositions)	Rich, nuanced and diverse, 43 primary *rationales* (e.g., setting a trap, deceiving a rival, exploiting rival weakness, preserving rival dignity)
Orientation toward action	Empirical examination of active behavior	Empirical examination of both active and passive purposeful behavior (i.e., actions and forbearances)
Strategic importance of tactics	Tactics are not strategic	Tactics can have long-term, broader, strategic roles in building resource advantages and maintaining strategic consistency

## Conceptual Development

### Prior Work and Orientations

Scholars of competitive dynamics have long sought to provide insights into firm rivalry by probing “the motivations and cognitions of actors who initiate and respond to competitive actions” (Chen & Miller, 2012). Most research has concentrated on conceptual models of how managers identify and process data about rival firms, with little information coming from the managers themselves. For example, early work applied a simple expectancy-valence framework to explain how the value of a reward and the probability of its attainment triggered competitive attacks and responses (Chen & Miller, 1994). Building on this work, some studies adopted the influential AMC model to explain behavior based on a firm’s *awareness* of a rival attack and its *motivation* and *capability* to respond (Chen, 1996). Critically, inferences associated with these qualities were drawn from properties of the actions themselves, such as complexity, irreversibility, timing, or strategic importance (Chen & MacMillan, 1992; MacMillan, McCaffery, & van Wijk, 1985; Smith et al., 1991).

Some studies found that managerial *awareness* was heightened when other firms were reputed market leaders or predators or when they possessed similar resources and markets in common (Chen, 1996; Gimeno & Woo, 1996; MacMillan et al., 1985; Smith, Grimm, & Gannon, 1992). It was also influenced by top team heterogeneity and information-processing capacity (Hambrick et al., 1996; Marcel, Barr, & Duhaime, 2010; Nadkarni & Chen, 2014; Smith et al., 1991). By contrast, action complexity and the use of vague language were argued to obscure intent, compromising awareness (Gao, Yu, & Cannella, 2016; Guo, Yu, & Gimeno, 2017). Interpretation was also shaped by managerial cognitive frameworks (Marcel et al., 2010) and temporal dispositions (Chen & Nadkarni, 2017; Nadkarni, Chen, & Chen, 2016).

Core contextual drivers of the *motivation* to engage included market centrality and identity domains (Chen & Miller, 1994; Livengood & Reger, 2010), prior interactions, tension between rivals, network embeddedness, resource similarity, and multimarket contact (Chen et al., 2007; Gnyawali & Madhavan, 2001; Kilduff et al., 2010; Young, Smith, & Grimm, 1996; Yu & Cannella, 2013).

Finally, a frequently cited stimulus for action was superior *capability*. A firm was found to act or respond when it had superior technological resources (Ndofor et al., 2011), network positions and alliances (Andrevski et al., 2016; Gnyawali & Madhavan, 2001), information-processing capacity (Chi, Ravichandran, & Andrevski, 2010; Smith et al., 1991), slack resources (Carnes et al., 2019), and scale (Chen & Hambrick, 1995).

Although this research provides important insights into the processes of attending and reacting to competitive information, our understanding of the reasons for managers’ competitive actions remains limited. Most research has tended to *infer* the reasons behind actions *indirectly* from the attributes or features of managers, firms, markets, and actions. What remain underexplored are managers’ own rationales that explain why actions are initiated at a specific time and in a specific context. In this study, we introduce the concept of competitive rationale and, based on our findings, develop a framework capturing and classifying key rationales that drive tactical actions.

### The Concept of Competitive Rationale

We leverage the causal theory of action to provide a theoretical foundation for competitive rationales (Davis, 2013; Davidson, 1963; O’Connor, 2013). The premise of the theory is that reasons underlie human behavior. The focus is on an agent’s *own* reasons for initiating an action rather than any external events that may stimulate an action (O’Connor, 2013). An agent has a reason to act when that action is a result of a *desire*—wanting to achieve something and a *belief*—confidence that by taking a specific action one would attain something desired (Davidson, 1963). Of course, reasons explain actions or deliberate inaction regardless of whether an agent’s belief is correct. Similarly, whether a reason adheres to certain conventions or norms is also not critical for it to drive an action. Thus, the focus of action philosophers is on *primary* reasons—*reasons that directly cause a specific action or inaction* (Davidson, 1963), regardless of whether they comply with norms or achieve agent goals (Davis, 2013).

Primary reasons describe what an agent wants to accomplish with an action (intent) and why it carries out that particular action (explanation) (Davis, 2013). “To know the primary reason why someone acted as (s)he did is to know an intention with which the action was done” (Davidson, 1963: 689). However, the concept of primary reason is broader than intention, comprising also explanation. “In explaining an action, we come to know why the agent acted as (s)he did; and to know why someone does something is to know the reason for his/her doing it” (Everson, 2013: 145).

The causal theory of action also implies the counterfactual. When action is said to occur because of a primary reason X, we also imply that it would not occur in the absence of X (Davis, 2013: 36). In other words, insufficient knowledge of the primary reasons for a specific action hobbles its interpretation. “When we ask why someone acted . . . we want to be provided with an interpretation. When we learn his/her reason, we have an interpretation . . .” (Davidson, 1963: 691). Hence, it follows that understanding such reasons is essential for explaining and predicting competitive behavior. We build on the causal theory of action to garner insight into the reasons that inform rivalry.

In this study, we focus on primary reasons that explain why an agent initiates a particular competitive action. For example, a coach can substitute players (an action) because they were not disciplined in executing a particular tactic (the primary reason). Of course, this action can serve other goals such as improving performance. However, the latter are not primary reasons for the substitution and have limited predictive utility—it is of little value to know opponents made a substitution to perform better. Primary reasons, by contrast, provide more *actionable* information. Knowing a rival’s action addresses a specific defensive failure can guide a coach toward a way to thwart that effort.

In short, we define competitive rationales as the primary reasons that directly explain why specific actions are initiated at a given moment and under specific circumstances. These rationales are conceptually distinct from less specific reasons explored in previous research, including superior capabilities, favorable market conditions, psychological states, and cognitive dispositions. The latter describe general conditions, dispositions, and goals that can trigger any action but do not explain why a particular action was initiated. We elaborate on these contrasts below.

### Competitive Rationales Versus Other Reasons Studied in Competitive Dynamics

#### Superior Capabilities

As noted above, a common reason for carrying out competitive actions is said to be a firm’s superior internal and external resources and capabilities (e.g., Andrevski et al., 2016; Carnes et al., 2019; Gnyawali & Madhavan, 2001; Ndofor et al., 2011; Smith et al., 1991). Clearly, however, capabilities are *not* primary reasons for carrying out a particular action. They reflect a general potential to act but do not explain the specific reason why firms initiate a particular action at a given time and situation.

#### Market Conditions

Other studies have explored the role of market characteristics, such as centrality, identity domain, alliance network position, and multimarket contact in explaining competitive actions and responses (Chen & Miller, 1994; Gnyawali & Madhavan, 2001; Livengood & Reger, 2010; McGrath et al., 1998; Yu & Cannella, 2013). Market conditions can trigger multiple types of actions but do not differentiate between their rationales. They only define a precipitating context but cannot explain just what managers want to achieve.

#### Subjective Perceptions of Competitors

Other reasons for acting have been inferred from psychological experiences of rivalry and competitive tension (Chen et al., 2007; Kilduff et al., 2010). Again, these factors can stimulate actions but do not explain why a particular action occurred. For example, competitive tension might motivate a firm to cut prices because of a cost advantage or to match a rival’s price cut. Alternatively, the firm might forbear from cutting prices in an effort to deescalate rivalry. Each of these rationales can be triggered by competitive tension. Thus, perceptions of rivals may tempt one to act, whereas primary reasons explain why an action occurred.

#### Managerial Cognition

Research on managerial cognition has examined factors that affect how executives notice and interpret rivals’ actions (Marcel et al., 2010). Action and top management characteristics, firm information processing capabilities, and managers’ cognitive frameworks affect how managers filter information, focus attention, and identify rivals (e.g., Hambrick et al., 1996; Nadkarni & Barr, 2008; Nadkarni & Chen, 2014; Smith et al., 1991). Such factors help to explain where managers direct their attention but not why they initiate a particular action. Noticing an action is quite different from knowing the reasons behind it. Like perceptions, cognitive dispositions are personal traits that predispose managers to take actions, but they do not explain why managers initiate a specific action.

### Why Study Competitive Rationales?

Whereas previous research examined *whether* a competitor is able and likely to initiate a competitive attack or response, our study asks *why* a competitor initiates a particular action or response and what it intends to achieve with it, thereby directly linking cause and effect. According to the causal theory of action, “the explanation by reasons is a type of causal explanation” (Davis, 2013: 35). When we know the primary reason that causes an action, we can understand what an agent wants to accomplish and why a competitive action is carried out. For example, by studying the primary reasons, we can learn whether an action is directed to neutralize a rival’s action or is primarily intended to address other organizational and social issues. We can also get a better understanding of the scope of its intended outcomes: Does it address the immediate situation or involve longer term objectives? Is it intended to deal with a current rival or primarily aimed at shaping relationships with other parties? Is it an integrative part of a broader strategy? Equally important, by focusing on primary reasons, we can distinguish competitive rationales from other reasons studied in previous competitive dynamics research.

Thus, in this study, we take initial steps toward developing a conceptual framework for studying competitive rationales. By leveraging an inductive approach, we surface key types and scope domains of competitive rationales that help to explain and predict tactical competitive behavior.

## Method

### Research Design and Setting

To gain insight into the rationales for tactical actions, we collected qualitative data from interviews and observations of basketball coaches. We employed a multiple-case study approach to analyze data from multiple decision makers on multiple occasions (Eisenhardt, 1989). Although findings from sports settings are sometimes thought to be limited in generality, they are appropriate for studying tactical decision making such as that by midlevel brand or sales managers. Moreover, their advantages of unambiguous observation, precise measures, motivated participants, and few confounds have for decades enlightened organizational analysis (Day, Gordon, & Fink, 2012; Wolfe et al., 2005). For example, studies of basketball and baseball provide insight into the nature of tacit knowledge, human capital, organizational status, strategy, and competitive advantage (Berman, Down, & Hill, 2002; Poppo & Weigelt, 2000; Sirmon, Gove, & Hitt, 2008; Washington & Zajac, 2005; Wright, Smart, & McMahan, 1995). Sports also are a common domain for competitive dynamics scholars, especially those pursuing sociocognitive aspects of rivalry (Kilduff et al., 2010; 2016; Miller, Pastoriza, & Plante, 2019; To, Kilduff, Ordonez, & Schweitzer, 2018). For example, Kilduff et al. (2010) demonstrate revealingly how institutionalized rivalries shape the intensity of basketball play and outcomes.

As with other sports-related studies, our choice of a basketball coaching setting afforded several advantages for studying tactical behavior. First, a game unambiguously consists of two head-to-head rivals competing with visible actions that require little time and resources to execute. Second, when games are in progress, coaches must consider both the short-term and long-term impact of their actions on their team’s capabilities, rivals, and stakeholders. Third, a member of our research team was granted proximate, complete, and unfiltered access to the primary reasons stated for each action or inaction during multiple games.

Basketball coaching also is a useful context for exploring tactical nuance and complexity. If tactical rationales are diverse, multifaceted, and potentially strategic within this temporally and resource-constrained context, these characteristics are apt to be even more prevalent in business, where managers have more time, resources, and competitive options to consider (see Table 2).

**Table 2:** Basketball Competition Versus Business Competition

	Basketball Competition	Business Competition
Action development time	Seconds to minutes	Months to years
Action development resources	Limited	Considerable
Competitive interaction duration	Periodic (quarter/game)	Open-ended
Decision makers	Few (head coach and two assistant coaches)	Several (top managers and middle managers)

#### Preliminary Interviews

We decided on a basketball coaching setting only after conducting preliminary interviews with seven coaches from seven basketball clubs competing in the International Basketball Federation (FIBA) leagues as well as Canadian and United States college leagues (one of the authors was a professional player for 11 years in FIBA). Our meetings in June and July 2013 assessed the prevalence of tactical actions in their decision-making. We found that *tactical forbearances—*that is, deliberately deciding *not* to act for specific reasons*—*occurred frequently. These were captured in statements made by the coaches we interviewed, such as “we wanted to X . . . but we decided to hold back because.” In this exploratory stage, we also assessed coaches’ readiness and capacity to provide rationales. Their responses were encouraging, prompting us to develop a protocol to conduct postgame interviews with coaches to capture the rationales for their tactical behavior during games.

### Data

#### The Sample

We relied on personal contacts with coaches and sports directors to contact the coaches of all 10 men’s teams of a European country’s first national league. Nine out of 10 coaches agreed to participate due to their familiarity with the first author. They were interviewed on more than one occasion as their teams participated in multiple games. This allowed us to collect data from multiple coaches and multiple games for each coach. In total, we conducted 30 postgame interviews with 9 head coaches about their tactical decisions in 15 basketball games in one European country (a member of the FIBA–Europe). We collected archival data for each coach and team from three sources: the statistical database of FIBA Europe, the basketball federation of the country in which we conducted interviews, and the videotaped games. Coaches, all male nationals, had diverse coaching experience (2-33 years), coaching success (0-6 championship titles), prior team rankings (from 2nd to 10th place in the previous season), and age (32-62 years).

We focused our data gathering on 15 regular season games taking place between October 19, 2013 and November 11, 2013. The set of games played on October 19 represented the second week of the 2013 to 2014 basketball season. We used the first week of games—played on October 12, 2013 but not included in our sample—to test our video equipment, check the quality of the edited videos, get feedback from the coaches about the videos and their availability for interviews, and inform them about the interview protocol using the videos.

#### Data Collection Procedures

The lead author conducted all interviews to reduce person-specific inconsistencies in the data collection process (Corley & Gioia, 2004). He was entirely familiar with basketball terminology and, again, had extensive professional basketball experience. Coaches were assured of confidentiality.

In collaboration with the basketball federation of the country, we hired a professional media firm to videotape each game using two camcorders: one to capture action on the basketball court and the other to capture information on the electronic scoreboard. The videos were then electronically spliced together to continuously show the game score, time period, and game clock time as the game was in progress. These videos were used in postgame interviews with the coaches to facilitate a retrospective analysis of the games and coaches’ decisions. The lead author met with each coach within a couple of days after the game—whenever the coach was available—but before his next scheduled game. Eighteen of the 30 interviews (60%) took place within two days of the focal game with another 5 interviews (cumulatively 77%) within 3 days of the game. The remaining 7 interviews occurred within 6 days of the focal game.

The interviewer presented the following script to each coach before the interview (also included in the consent form):

*As a basketball coach, you make many decisions during a basketball game, such as decisions to substitute a player, not take a timeout, change a play, or continue playing the same defensive formation. Thus, you are making decisions to act or not, for example, because you believe not changing would improve your team’s performance.* ***I will ask you to elaborate on the reasons why you chose to act or not to act at that time.*** *I ask you to comment on all decisions you made during the game: decisions to act and decisions to purposefully not initiate a considered action.*

Thus, our focus was on exploring the rationales—the primary reasons—for considered tactical actions or forbearances. We conducted interviews using two laptop computers: one for watching the game and one for taking notes. The coach was instructed to stop the video at any point during the replay of game when he *considered* a tactical move—whether carried out or not—such as changes in offensive or defensive tactics, player substitutions, and timeouts. Interviews lasted from 2 hours and 10 minutes to 2 hours and 55 minutes per game. As the interview progressed, the first author noted, in an Excel spreadsheet, critical facts associated with each coach-initiated stoppage of the video playback: the type of tactical move considered, the time and period of play displayed on the game clock, and the score of the game at that moment. Most importantly, for each coach-initiated video playback stoppage, the interviewer wrote verbatim the coach’s detailed reason for carrying out—or not—the tactical action considered at that precise time. Table 3 presents numerous examples of various rationales translated from the coach’s native language.

**Table 3:** Types of Action Rationales (AR) and Forbearance Rationales (FR)

	Examples of Coach Rationales	Primary Rationales	Second Order Categories	Rationale Types
AR1	**N/A**	Wait and see	To learn	Organizational rationales
FR1	We did not play well man to man defense. I started thinking of changing to zone defense. But I thought it was too early; I waited a little bit to see if it improves.			
AR2	**N/A**	To preserve an option		
FR2	It was a bad start of the game. I thought about taking a time out because we had 6 unanswered points. I did not want to start lagging behind too much and not being able to catch up later especially because we played at the opponent’s home court. However, I still decided to not take time out to preserve it for later.			
AR3	He did not box out, so I take him out for a mistake in defense. I wanted them to know that there will be no tolerance for such mistakes.	Team learning		
FR3	[Player X] and [Player Y] are new additions to our team; this is their first game and they are still not well synchronized with the other players; I thought of substitution, but I delayed this decision to give them an opportunity to learn how to play with the other players; our chances to win this game were very low, so I was OK with taking this risk.			
AR4	[Player X] enters the game. He is a young player who needs opportunities to play. I wanted also to assess how much he is currently able to help us in the future games.	Player learning		
FR4	I thought about replacing [Player X]; however, I decided to keep him because he needs minutes to get back in shape.			
AR5	I introduce zone defense for two possessions to see if it works and whether I can use it later. The score was comfortable, so I can test whether zone could be effective if they make a comeback later in the game.	Coach learning		
FR5	I thought about changing my zone defense. . . . However, I decided to stay with the same defensive strategy because I strongly believed that given our players, this type of defense is ideal. . . . It will hurt us a little bit today, but I am OK with that.			
AR6	I replaced [Player X] with a fresh player to increase the intensity in defense; I replaced [Player X]; he did not play well and the game is on the line now. I needed boost from the bench.	To boost energy	To adjust strategy	
FR6	**N/A**			
AR7	**N/A**	To preserve strategic consistency		
FR7	I noticed their big guy is in foul trouble, so I thought we might exploit that advantage. Our center can attack him and draw a foul. However, our strategy worked well, we moved the ball well, and this move might slow the ball movement. So I decided to not change anything.			
AR8	I am taking out [Player X] and putting a better offensive player. He made a mistake and it was a close game. I had to prevent other mistakes; the current score gives us a good chance to win the game.	To fix execution failures		
FR8	I decided not to change how we defended their shooters although they just scored another 3-point shot. I believe that my strategy will work so I told them to keep guarding “under screen.”			
AR9	I am changing the way we guard pick & roll - from “switching” to “help & recover.”	To change strategy		
FR9	**N/A**			
AR10	I am taking out two players, to prevent possible injury. Also, we have an important game in three days, so they need rest.	To preserve human resources	To orchestrate resources	
FR10	I thought of putting [Player X] in to improve rebounding. However, I would risk getting him in foul trouble because he would need to guard a smaller and faster player. So, I restrained from this action.			
AR11	I am replacing [Player X] with [Player Y]. [Player X] does not play hard defense because he has 3 fouls.	To optimize resources		
FR11	I thought about putting [Player X] back in the game. However, I decided to give him more rest and also to keep him out of foul trouble because his replacement was doing a good job.			
AR12	I started playing pick & roll on the top. It was pick & pop actually, because we did not have classic center. Our centers are great shooters and we can exploit this advantage now.	To exploit a superior resource		Organizational rationales
FR12	**N/A**			
AR13	I made substitution because of injury of another player.	Forced move	To overcome strategic limitations	
FR13	**N/A**			
AR14	**N/A**	Lack of options		
FR14	I thought about changing to “match-up” zone [defense]. However, our team is young and match-up zone is inappropriate for these players, so I kept playing 2 to 3 zone defense.			
AR15	**N/A**	Lack of resources		
FR15	Our defensive rebounding was a big problem. I made substitutions earlier to remedy this problem, but the problem still exist. At this point, I do not have quality players to fix this problem.			
AR16	**N/A**	Inadequate resources		
FR16	I thought the best way to defend was to put match up zone; however, our team is young and I was not comfortable with it. Match up zone is inappropriate for these players, so I stayed with the previous zone defense.			
AR17	**N/A**	Lack of time		
FR17	I wanted to take [Player X] out. However, there was no opportunity to make substitution. (No game interruption.)			
AR18	**N/A**	To adjust to unanticipated change in situation		
FR18	I am thinking of taking a timeout because the ball was not moving quickly. However, the opponent missed open shots, and I decided to not take a timeout. I tried to fix the offense issues with instructions from the bench.			
AR19	I changed a play without taking a timeout. We are playing long plays to spend time.	To appropriate a rival’s resources	To preempt	Rivalrous rationales
FR19	**N/A**			
AR20	I told my players to make fouls now because we were not in bonus. I wanted to reduce the time for executing their last possession.	To preempt a rival		
FR20	I am not taking a timeout here even though I need to talk with my players about several defensive errors. However, the opponent does not have other timeouts left, so I did not want to use my timeout to advise his players. The score is going in our favor.			
AR21	I faked playing zone defense. Our players make zone defense formation, but after their first pass, we actually play man-to-man defense.	To mislead a rival	To deceive rivals	
FR21	I thought of modifying our defense. However, I decided to not fix it during the game because we were losing this game anyways. We will fix the issue in practice. I want to trick our next opponents into believing they can easily attack our defense.			
AR22	We started fouling to get faster in bonus, so we can stop the game by giving the opponent free throws. This way we can get more possessions before the end of the game and get more opportunities to cut their lead. We also needed to score quickly in offense.	To set a trap		
FR22	**N/A**			
AR23	I am keeping my players 30 sec more on timeouts despite referees’ warnings. I play with only 6 quality players and they needed rest.	To bend rules		
FR23	**N/A**			
AR24	I switched to man-to-man defense to avoid being predictable. They had time to prepare for my zone defense on half time, so this would surprise them.	To surprise a rival	To surprise	
FR24	I wanted to change to zone defense . . . however, I postponed this action for the second half, so the opponent will not have time to prepare during the halftime break how to attack the new zone defense. I was pretty sure they did not expect us to play 1-3-1 zone defense.			
AR25	I have only 6 seconds for this possession. It is very important to score, so I draw a play to score from inbound.	Strategically timing a competitive move		
FR25	**N/A**			
AR26	I am asking again our shooting guard to organize our offense because our point guard was pressed heavily.	To neutralize a rival’s action	To neutralize a rival’s advantage	
FR26	**N/A**			
AR27	[Player X] is in; I am playing with my best 5 players, because the zone defense of the opponent is creating problems for us.	To neutralize a rival’s defensive strategy		
FR27	**N/A**			
AR28	I put zone 1-3-1 because we were not able to stop their pick & roll. Zone defense can neutralize that.	To neutralize a rival’s offensive strategy		
FR28	**N/A**			
AR29	[Player X] is in. I needed to stop their best player and [Player X] is good defender	To neutralize a rival’s advantage		
FR29	I wanted to take [Player X] out. However, they made substitution and I thought [Player X] will match well with their new player. So, I decided to keep him in the game.			
AR30	They made run, so I had to stop them. It is a great team, so if you let them get away it will be tough to catch up . . .	To disrupt a rival’s rhythm	To disrupt rival momentum	
FR30	**N/A**			
AR31	I changed again to zone 2-3 (from 1-3-1); I just wanted to interrupt their offensive game; I did not want them to adjust to 1-3-1 zone.	To disrupt a rival’s strategy		
FR31	**N/A**			
AR32	I got into argument with the referees and risked getting a technical foul to create tension.	To create psychological advantage		
FR32	**N/A**			
AR33	They took their center, [Player X], out of the game. I started posting their big guys to take advantage inside.	To exploit a rival’s weakness	To attack rival weakness	
FR33	**N/A**			
AR34	I replaced a guard with a power forward. We wanted to put their bigs (centers, power forwards) in foul trouble.	To create a rival’s weakness		
FR34	**N/A**			
AR35	I make fast substitutions to provide opportunity for more players to “feel” the game; to stay motivated so I can count on them in the second half.	To motivate	To manage team dynamics	Social rationales
FR35	I am thinking of replacing [Player X]. But . . . he is negatively affected when taken out early after an error.			
AR36	I am putting [Player X] in. He needs to play more to regain his confidence.	To increase player confidence		
FR36	[Player X] got into a series of mistakes. I am thinking of substituting him, but I wait to give him a chance to make some shots and regain confidence.			
AR37	I replaced [Player X]. He was not organizing the play well.	To improve team coordination		
FR37	**N/A**			
AR38	We missed “bunny” [an easy shot]. I took a timeout to settle down my team and better control the score at the end of the game.	To increase team composure		
FR38	**N/A**			
AR39	**N/A**	To sanction behavior		
FR39	“[Player X] was unhappy with my decision to replace him earlier. I was thinking of putting him back in the game now, but because of his reaction earlier, I decided to not play him anymore.			
AR40	I put [Player X] back in the game. . . . The game was already decided, but I wanted to cut the lead to not lose too much.	To preserve team dignity & reputation		
FR40	I am thinking of putting our young players in the game to gain experience. We will lose this game anyway. However, I still try to keep the score difference in a reasonable range; it is embarrassing to lose by a fifty-point differential.			
AR41	I put a young player because we were up 35, so they can reduce our lead. I did not want to be perceived by my opponent as disrespectful.	To preserve rival dignity & reputation	To manage relationships with other parties	
FR41	I wanted to take timeout. There were many loose passes, errors. I also thought of taking out our best players to prevent injuries. However, I did not take any action because of collegiality and showing respect to the other coach; I was up 30 and calling a timeout would be disrespectful.			
AR42	I argued with the refs. I think that pressure on the referees resulted in a couple of decisions in our favor later in the game.	To manage relationships with referees		
FR42	**N/A**			
AR43	I talked to fans to calm down a bit. Referees warned me that they would empty the building if they continue making problems (throwing things on the floor). The referees did not empty the building, which helped us win this game.	To manage relationships with fans		
FR43	Referees wanted to empty the building; fans were throwing parts on the court; I let one of my players talk with fans.			

Our study employed retrospective interviews with coaches as they watched videos of their own games. Thus, tactical actions and rationales may have been underreported due to incomplete or inaccurate coach recollections. We tried to limit such recall bias by conducting interviews soon after a game. Also, our protocol never broached issues of the success or appropriateness of moves.

### Analysis

Our data include 841 statements from coaches transcribed during the interviews describing their reasons for acting or not acting. Two authors with limited basketball knowledge and not involved in the data collection process coded the data together with the expert author who had collected that data (samples of the raw data are shown in Table 3). During the analysis, the expert-author described and clarified the meaning of any “technical” terms used by the coaches (e.g., zone press, pick-and-roll, pick-and-pop, switching in defense, boxing out, etc.). Coding was conducted collectively using both the lead author’s expert interpretations and the other two authors’ interpretations of coaches’ statements.

In this “first-order” analysis, the three coders attempted to identify from coaches’ statements all primary reasons for actions or inactions, proceeding until saturation was reached. This was an inductive iterative process involving two day-long in-person discussions among three authors (Grodal, Anteby, & Holm, 2021). The authors generated labels for the rationales they believed were embodied in the coach statements, keeping the labels as close as possible to the coaches’ terminology—for example, to surprise a rival, to preempt a rival, to enhance player learning, to increase player confidence, or to improve strategy execution (Gioia, Corley, & Hamilton, 2013). Saturation level was reached after 300 analyzed statements, which covered the first five games. In fact, after the first 250 statements hardly any new rationales emerged. This analysis yielded 34 primary rationales for tactical actions and 24 for tactical forbearances (with some actions and forbearances having rationale types in common, yielding 43 rationales in total). Table 3 illustrates the hierarchical structure of our data, including translated representative coaches’ statements for each of the 43 primary rationales.

In a second step, we searched for similarities across primary rationales and attempted to group them into more general, second-order categories (Grodal et al., 2021). To accomplish this reliably, two authors independently analyzed similarities among primary rationales and attempted to group them into more general categories (e.g., to learn, to adjust strategy, to orchestrate resources, to deceive rivals, to manage interpersonal relationships). Next, the three authors met to reconcile the categories, reaching agreement on 12 of them (in 3 cases where there was disagreement among the raters, separate categories were preserved). The expert author then went back to code *all* 841 statements. To establish coding reliability, another author blindly coded a random sample of a different set of 42 statements (5% of the data). The resulting Perreault and Leigh’s (1989) reliability index was 0.74 for second-order categories, above the prescribed 0.70 cutoff.^1^

In revisiting statements within our primary rationales and their second-order categories, and through more discussion among the authors, we identified three overarching third-order rationale types. These are rivalrous, organizational, and social rationales. *Rivalrous* rationales prioritized a current and specific rival and its actions. Organizational rationales were primarily directed toward making organizational adjustments such as preserving resources, fixing execution failures, or maintaining strategic consistency. Finally, *social* rationales prioritized clearly relational and interpersonal issues—for example, to motivate, avoid embarrassment, or preserve rival dignity.

Within each of the three rationale types—rivalrous, organizational, and social—the three authors as a group revisited the primary rationales to discover the *scope of their target domains*, the second dimension of our framework (Tavory & Timmermans, 2014). Rationales were found to vary according to temporal and strategic scope and the range of external parties considered beyond the current rival. This process identified three target domains—the immediate situation, longer term strategic outcomes, and other external parties. Together, the three rationale types, each reflecting three potential target domains, constitute the structure of our findings (9 cells, see Table 4). We classified the primary rationales accordingly. The authors reached consensus on most of the rationales identified by a unique cell in Table 4 (I to IX). In eight cases, the primary rationales were allocated to two cells.^2^

**Table 4:** Conceptual Framework for Studying Competitive Rationales

Scope of Rationales	Types of Rationales		
	Rivalrous	Organizational	Social
Immediate situation (immediate rival, actions, and outcomes)	(I)	(IV)	(VII)
	To preempt a rival	To boost energy	To increase team composure/coordination
	To neutralize a rival’s action	To fix execution failures	
	To neutralize a rival’s advantage	To exploit superior resources	To sanction behavior
	To appropriate a rival’s resource		
	To disrupt rhythm		
	To set a trap		
	To surprise		
	To mislead		
	To defy/bend rules		
	Timing a move		
	To create/exploit rival’s weakness		
Longer term strategic outcomes (longer term vs. immediate effects, and overall strategy vs. single tactical action)	(II)	(V)	(VIII)
	To surprise *current rival* in *future* game periodsTo disrupt rival strategyTo neutralize rival strategy	Team learningPlayer learningCoach learningTo wait and seeTo preserve optionTo preserve resourcesTo optimize resource usageTo change strategyTo preserve strategic consistency	To motivate players/teamTo increase player confidenceTo preserve team dignity/reputationTo preserve *rival* dignity
External parties beyond a direct rival (other external stakeholders vs. a direct opponent)	(III)	(VI)	(IX)
	To mislead and conceal a move from *future rivals*	To develop resources for competing against *future rivals*	To calm *fans*
			To influence *referees*
	To create psychological advantage by getting in argument with *referees*		

## Findings

### Types of Competitive Rationales

Whereas all tactical actions are competitive—that is, intended to improve performance vis-à-vis rivals—their primary reasons can be rivalrous, organizational, and/or social. *Rivalrous* rationales are reasons with a primary purpose, for example, to outwit competitors, disrupt rival rhythm, mislead current and future rivals, or neutralize rival strategy. *Organizational* rationales are reasons related to organizational adjustments. Although these rationales can ultimately affect performance and thus rivals, their primary intent is to address the organization itself, not rivals or their actions. Finally, *social* rationales deal primarily with relational and interpersonal issues. They address norms and expectations of various stakeholders and manage team dynamics.

Even in a highly competitive zero-sum situation, 74% of all rationales mentioned by coaches sought to improve the general capacity to compete through improved *organization*. For example, they substituted players not to attack or respond to an opponent but primarily for organizational reasons such as nurturing resources and ensuring strategic coherence. Three hundred and fifty (42%) rationales related to adjusting strategy, 213 (25%) to organizational learning and orchestrating resources, and 58 (7%) to dealing with strategic limitations. Social rationales surfaced here, too, in maintaining morale and confidence and protecting the dignity of opponents and others. Twenty-three nonrivalrous rationales were initiated for social reasons (3%), while the rest were rivalrous (23%).

Competition in business, too, is not simply a matter of responding to rivals or attacking them. Firms must build organizationally and socially while in the heat of competition in order to become more effective. For a responder to erroneously assume that an action is backed by rivalrous intent can lead to retaliatory responses that unnecessarily escalate rivalry.

### Scope of Competitive Rationales

Our second dimension considers how rivalrous, organizational, and social rationales differ in scope across the three target domains of *immediate situation*, *long-term strategic outcomes*, and *other external parties.* As noted, Table 4 juxtaposes the three rationale types and three target domains of rationales. Each cell in the table embeds the primary rationales that most closely correspond to each rationale type and target domain.

The first target domain—the immediate situation—describes rationales primarily concerned with a direct rival, action, and its immediate effects (within the same quarter of the game or a single exchange). Typically, these rationales aim to neutralize a rival’s specific action, exploit sudden opportunities to score, and prevent execution failures. The second domain comprises rationales with a longer term and strategic orientation. They seek benefits over multiple exchanges and periods of the game and possibly even beyond a current game; they mainly focus on achieving broader, strategic purposes such as disrupting rival strategy, testing and modifying one’ own strategy, and protecting reputation. The third domain captures rationales that deal with a team’s external parties other than a direct rival, which include future rivals, fans, and referees.^3^

The types and scope of rationales are common for both actions and forbearances. Coaches often used forbearance for rivalrous, organizational, and social reasons—they were especially instrumental for achieving longer term and systemic/strategic goals. We identified 673 rationales for action (80%) and 168 for forbearance (20%) (see Table 5).

**Table 5:** Competitive Rationales by Actions and Forbearances

Rationale Categories				Action	Forbearance		Total	Percentage
Organizational				477	144		621	74
To adjust strategy				312	38		350	
To learn				52	55		107	
To orchestrate resources				87	19		106	
To overcome strategic limitations				26	32		58	
Rivalrous				183	14		197	23
To attack a rival’s weakness				64			64	
To deceive rivals				8	5		13	
To disrupt rival momentum				35	1		36	
To neutralize a rival’s advantage				49	3		52	
To preempt a rival				11	3		14	
To surprise				16	2		18	
Social				13	10		23	3
To manage relationships with other parties				7	5		12	
To manage team dynamics				6	5		11	
Grand total				673	168		841	100
Coach ID	Number of Rationales			% Forbearances	Coach Age	Headcoach Experience	Championships Won	Runner Up
	Action	Forbearance	Total					
Coach 1	81	35	116	30	62	33	6	5
Game 1	25	17	42	40				
Game 5	26	9	35	26				
Game 11	30	9	39	23				
Coach 2	62	12	74	16	40	4	1	1
Game 1	21	8	29	28				
Game 6	18	2	20	10				
Game 9	23	2	25	8				
Coach 3	51	2	53	4	44	3	0	1
Game 2	15	1	16	6				
Game 8	16		16	0				
Game 15	20	1	21	5				
Coach 4	105	18	123	15	40	4	0	0
Game 2	27	5	32	16				
Game 7	24	4	28	14				
Game 11	27	4	31	13				
Game 14	27	5	32	16				
Coach 5	80	14	94	15	47	16	1	3
Game 3	19	4	23	17				
Game 6	17	3	20	15				
Game 10	18	1	19	5				
Game 14	26	6	32	19				
Coach 6	68	19	87	22	44	4	0	1
Game 3	17	8	25	32				
Game 9	22	8	30	27				
Game 13	29	3	32	9				
Coach 7	102	28	130	22	32	2	0	0
Game 4	26	16	42	38				
Game 7	26	7	33	21				
Game 12	30	2	32	6				
Game 15	20	3	23	13				
Coach 8	83	16	99	16	44	5	0	1
Game 4	23	9	32	28				
Game 5	20	2	22	9				
Game 10	24	4	28	14				
Game 13	16	1	17	6				
Coach 9	41	24	65	37	36	6	4	2
Game 8	19	21	40	53				
Game 12	22	3	25	12				
Grand total	673	168	841	20				

### Competitive Rationales Framework

#### Rivalrous Rationales

As noted, rivalrous rationales are directed toward acting or responding directly to opponents. Their target domain may be the immediate rival and outcomes, longer term strategic moves directed to rivals, and other external parties in the competitive exchange.

##### Immediate situation

Given the traditional focus of the competitive dynamics literature, it was not surprising that many actions were rivalrous; that is, intended to outmaneuver opponents at a given moment in time. The first set of rivalrous rationales (Cell I in Table 4) deals with direct rivals and immediate action outcomes. Some of these rationales have to do with surprising, confusing, and deceiving an opponent. Coaches deployed tactics to throw rivals off their game, specifically, to disrupt their rhythm (Table 3—AR30), set a trap (AR22), surprise them (A24; F24), or mislead them (AR21; FR21).^4^ For example, a coach attempted to negate rival momentum by telling players “*to make quick non-shooting fouls*” (Coach 2, Game 1 (C2, G1) in our data). Another coach took a timeout to disrupt his rival’s rhythm: *They scored several unanswered points. I took a timeout to stop their run* (C5; G3). Coaches also initiated actions to set a trap for the opponent: *We started fouling [in the last two minutes of the fourth quarter] to get faster in bonus, so we can stop the game by giving the opponent free throws. This way we can get more possessions before the end of the game* (AR22). One coach unexpectedly changed his defense after a timeout to surprise the opponent: *I changed to zone defense after a timeout; I wanted to surprise them* (C8; G4). Another coach attempted to surprise more regularly: *We alternate every other possession between zone and man-to-man defense to confuse them and stay unpredictable* (C6; G9).

Coaches also attempted to deceive opponents: *There were 2 seconds left. I called Player X to come talk to me. I told him to stay and pretend as if he is talking to me. Then, I told him to suddenly sprint and try to score by surprise* (C9, G12); they also intended to mislead with forbearance: *I faked playing zone defense. Our players made a zone defense formation, but after their first pass, we actually played man-to-man defense* (AR21). A coach even attempted to avoid strictly following the rules: *I am keeping my players 30 seconds more on timeouts despite referees’ warnings. I play with only 6 quality players and they needed rest* (AR23). Other rationales were more typical of prior work in competitive dynamics. For example, coaches initiated actions and forbearances to preempt rivals (AR20; FR20) and to neutralize their actions (AR26) and advantages (AR29; FR29) (e.g., Chen & Miller, 2012; Ma, 1999; Porter, 2008).

In summary, this set of rivalrous rationales primarily deals with the current and immediate competitive situation using a variety of tactical actions to outmaneuver opponents.

##### Longer term strategic outcomes

Other rivalrous rationales focused on achieving benefits *beyond the immediate situation and beyond single moves to achieve longer term strategic goals* (Cell II, Table 4). For example, we identified forbearances that were initiated to achieve longer term benefits, such as preserving moves to use as surprises later in the game: *I anticipated that the opponent would instruct his team how to attack 2-3 zone during the time out, so my intent was to surprise him with 1-3-1 zone defense. So I postponed this action to the second half* (C1; G5; FR24). Another coach identified a problem but decided not to address it with a contemplated action: *I feel that our man to man defense is weak. I am thinking now of trying a new type of defense (i.e., matchup zone), but I decided not to because I wanted to keep this defense as a surprise later* (C1; G1). The rationales for these forbearances go beyond creating short-term advantages to achieve a stronger position in the future.

Other competitive actions were initiated to influence a rival’s overall strategy rather than address any particular move. For example, a coach initiated a move to interrupt a rival’s strategy: *I changed again to zone 2-3 (from 1-3-1); I just wanted to interrupt their offensive game; I did not want them to adjust to 1-3-1 zone* (C1; G5). A coach also attempted to neutralize an opponent’s defensive strategy: *They were playing zone defense; I quickly reacted telling our team to change our offense* (C4; G2).

##### Other external parties

Rivalrous rationales concern not only current rivals but also other parties involved in a current or future competitive context (Cell III, Table 4). For example, some coaches used forbearances to conceal their intentions from *future* rivals: *I decided not to fix (a defensive error) because we were losing this game anyways. We will fix the issue in practice. I want to trick our next opponents into believing they can easily attack our defense* (FR21). One coach intentionally argued with the referees to increase competitive tension and create psychological advantage over an opponent: *I got into an argument with the referees and risked getting a technical foul to create tension* (AR32).

Collectively, the above statements make clear that although rivalrous rationales were primarily intended to outmaneuver current rivals, they differ in short-term versus longer term objectives, specific moves versus systemic strategic ones, and a focus on direct rivals versus other parties. This signals a range, complexity, and subtlety in rivalrous rationales hitherto unanticipated in the literature.

##### Business relevance

Our exploration of rivalrous rationales has surfaced neglected issues that challenge managers’ ability to interpret the competitive landscape. These include purposeful deception and forbearance, systemic and strategic timing of moves, considerations regarding *future* competitive engagements, and dealing with multiple stakeholders beyond a single rival. Clearly, in business situations where timing, disruption, and surprise are of the essence, these factors are likely to figure prominently.

#### Organizational Rationales

Organizational rationales are directed primarily towards improving team resources, strategy, and execution. As with rivalrous rationales, their target domains may be the immediate competitive situation, longer term strategy-related organizational moves rather than singular moves, and other parties.

##### Immediate situation

The first set of organizational rationales were primarily concerned with quickly fixing internal failures in execution, intensifying effort, and exploiting resources (Cell IV, Table 4). For example, some coaches addressed immediate issues with execution: *I took a timeout because of a bad pass. Our transition defense was also bad, we needed to fix this* (C3; G2). *I replaced [Player X]; he did not do a good job in defense* (C7; G4). *I took a timeout; we must move the ball faster. . .* (C2; G1). Some actions were initiated to bring new energy: *I put [Player X] in. I wanted to refresh my team, to bring more energy* (C1; G5). *I started playing full court pressing to try to reduce their lead* (C4; G2). *I put [Player X] in to enhance our offense; he is a very athletic player who can also help us with rebounding in defense* (C5; G3). Other actions were intended to exploit a superior resource: *I started playing ‘pick & pop.’. . .Our centers are great shooters and we can exploit this advantage now* (AR12).

##### Longer term strategic outcomes

Other organizational rationales were concerned with longer term outcomes (Cell V, Table 4). For example, some actions were initiated primarily to learn through experience: *I put [Player X] back in the game. I wanted to give him a chance to play more, so I will know whether I can count on him for other games and which of his qualities can help my team* (C1; G1). Another coach chose to forbear for similar reasons: *I am thinking of taking a timeout but I will wait because I want my players to struggle and learn how to deal with adversity* (C1; G1). This experiential process was initiated to gain longer term benefits, sometimes even beyond the current contest.

Forbearances, quite common in this category, were also employed when coaches wanted to reduce uncertainty by using a wait-and-see approach: *I thought about making a sub but waited a little bit because the score was favorable* (C2; G1). *We committed two errors; I am thinking of taking a timeout to prevent further score decline. But I will wait two more attacks* (C8; G4). At other times, forbearances were intended to preserve options: *I needed to call a timeout, but I decided to keep it for my last two attacks, to organize them, so we can finish successfully* (C7; G4).

Furthermore, coaches regularly initiated actions with a primary purpose to optimize resources (AR11): *I took [Player X] out, [Player Y] in. I just wanted to keep both players in rotation* (C6; G3). Other coaches stated: *I replaced [Player X] and [Player Y]. I wanted to rest players and put fresh players in* (C6; G9). Coaches also acted to preserve players for more opportune moments: *I replaced Player X . . . he was in foul trouble and I wanted to save him for the 4th quarter (C6; G13).* Forbearances were also intended to preserve resources (FR10; FR2): *I thought of putting [Player X] to improve rebounding. However, I would risk getting him in foul trouble because he would need to guard a smaller and faster player. So, I restrained from this action* (C9; G8). Another coach preserved an option to use a timeout at a later time: *I decided not to take a timeout to preserve it for later [when our team needs to rest]* (C4; G2).

Some actions were initiated to change strategy (AR9), *We change our defense from “hedging” to “switching” pick & roll* (C7; G7), and make strategic adjustments (AR8), *I took a timeout. Our offense was stagnant and I wanted to speed up the game* (C5; G10). Other coaches said they *avoided* taking actions that could slow execution: *I noticed their big guy is in foul trouble, so I thought we might exploit that advantage. However, our strategy worked well, we moved the ball well, and this move might slow the ball movement [killing our momentum]. I decided to not change anything* (FR7). They also prevented clashes with strategy: *I recognized an opportunity to exploit a mismatch. . . . However, I decided to not do that because I did not want my players to get out of our system [of play] . . . to start playing individually* (C7; G4).

Finally, actions were carried out to test strategy: *I put in zone defense for two possessions to see if it works and whether I can use it later. The score was comfortable, so I can test whether zone could be effective* (AR5). Similarly, one coach tried out a new defense in a game situation: *I changed into zone defense; we just want to try how it will work; there is not much time left in the game to hurt us, but we can learn from it* (C6; G3).

##### Other external parties

Beyond the current game, coaches initiated actions and forbearances to improve their team’s capabilities for competing against future rivals (Cell VI, Table 4). For example, a coach made substitution to test whether a player can be effective with the next opponents: *I put [Player X] back in the game. I wanted to give him a chance to play more, so I will know whether I can count on him for the next games and which of his qualities can help my team* (C1; G1). Another coach used forbearance to integrate new players in the team: *Player X and Y are new additions to our team . . . not familiar with our defensive schemes . . . not synchronized with the other players. I thought of substitution, but I give them opportunity to learn how to play with the other players. Our chances of winning this game are very low . . . I am thinking about the next opponent* (C1; G1).

Collectively, organizational rationales varied in their orientation to orchestrate resources, create learning opportunities, or make strategic adjustments. In the short run, some rationales addressed execution failures and increasing team intensity. In the long run, other rationales focused on real-time learning, preserving options, and optimizing resources. Not surprisingly, rationales for crafting strategy had a longer term orientation. Additionally, there were several rationales related to organizational limitations such as forced substitutions of players due to lack of time (FR17), injuries (AR13), or inadequate skills (FR14, FR15, FR16). We do not include those in Table 4 because they indicate involuntary decisions rather than purposeful actions or forbearances.

##### Business relevance

Competitive advantage is achieved, in part, through a firm’s ability to develop, nurture, and leverage superior resources. Conventional wisdom holds that managers develop resources primarily through strategic investments. Organizational rationales, by contrast, highlight the role of tactical competitive moves in enhancing current and longer term organizational capabilities. Indeed, rationales involving pacing and resource management, enduring consequences of engagement, parties beyond an immediate competitor, and the subtle orchestration of moves are likely to be critical in today’s complex, dynamic business context.

#### Social Rationales

Social rationales are directed toward improving interpersonal relationships with internal (e.g., players) and external parties (e.g., rivals, fans) to enhance competitive effectiveness. Again, its target domain may be the immediate competitive situation, longer term strategic outcomes, and external parties.

##### Immediate situation

Some rationales were aimed at quickly dealing with players’ appropriate or inappropriate behavior (Cell VII, Table 4). For example, one coach attempted to improve the immediate competitive position by increasing player discipline: *I tried to settle down my team; regardless of the score, we must play in a disciplined manner* (C2; G9). Another coach dealt directly with players’ performance by sanctioning them: *I took Player X out [because] he did not follow instructions* (C1; G11). *I did not want [Player Y] to be in the game at all; I was very mad at him!* (C4; G14). Social rationales also dealt with increasing team composure or coordination: *We missed “bunny” [an easy shot]. I took a timeout to settle down my team and better control the score at the end of the game* (AR38).

##### Longer term strategic outcomes

Cell VIII in Table 4 illustrates rationales primarily intended to motivate players, increase their confidence, and preserve team dignity that could improve team performance beyond a current game and immediate action (AR35, 36, 40; FR35, 36, 40). For example, a coach stated: *Player X needs rest, whereas Player Y is an older player, I want [Y] to feel involved in the game* (C6, G3); the same coach made another substitution: *I am putting [Player X] in. He needs to play more to regain his confidence* (C6; G3). Other coaches used sanctions to set an example and as a socialization tool: *I took [Player X] out for a mistake in defense. I wanted [my team] to know that there will be no tolerance for such mistakes* (C7; G4). Another coach chose forbearance for a similar reason: *[Player X] was unhappy with my decision to replace him earlier. At this time, I was thinking of putting him back, but because of his reaction earlier, I decided to not play him anymore* (C6; G13). Finally, coaches’ rationales considered other strategic outcomes such as preserving team reputation: *I put [Player X] back in the game. . . . The game was already decided, but I wanted to cut the lead to not lose too much [in the eyes of fans]* (AR41).

##### Other external parties

Coaches also actively managed other parties (Cell IX, Table 4). For example, one coach acted to calm fans (AR43), *I talked to fans to calm them down a bit* (C9, G8), and influence referees (AR42), *Several times I argued with referees about calling a ‘moving screen’* (C4, G2). Another coach took action to protect rival dignity and maintain a positive relationship: *I put a young [inexperienced] player because we were up 35 points, so they (the opponent) could reduce our lead. I did not want to be perceived by my opponent as disrespectful* (C6; G3).

Collectively, social rationales focused mostly on managing interpersonal relationships and team dynamics. In the short run, coaches acted to enhance team composure and sanction behavior, managing morale and team socialization. In the long run, they were more focused on motivating players and dealing with high-level strategic issues related to preserving team and rival reputation. They also managed other parties such as future rivals, fans, and referees.

##### Business relevance

Beyond the challenges associated with head-to-head rivalry, managers in business also need to be responsive to a wide range of stakeholders, such as suppliers, regulatory bodies, labor unions, environmental and social advocacy organizations, and the communities in which the firm operates. Our findings on social rationales suggest that managers carry out some tactical actions to address the complex motivations and emotions of salient social actors even in situations with high levels of competitive pressure. These social rationales therefore carry potential relevance to both the study and practice of competitive dynamics.

## Implications

The theory of causal action (Davis, 2013; Davidson, 1963; O’Connor, 2013) represents a useful foundation for assessing the human deliberations underlying competitive behavior to get at its underlying causes—that is, the primary reasons for why an action does or does not take place. It extends competitive dynamics beyond external cues from markets and firms to explain competitive behavior, deepening our understanding of *how* competitors behave and *why* they act or forbear in specific situations.

We find that competitive behavior involves an astonishing variety of primary reasons for the same types of tactical actions. Specifically, our basketball coaches considered *four* observable types of competitive actions: player substitution, timeout, defense change, and offense change. By contrast, as noted, we identified 43 primary rationales, many of which could drive a single type of action. For example, Table 3 shows the many rationales motivating player substitution.^5^ In addition, rationales were often intended to mislead, surprise, or forbear, rendering them potentially inscrutable. Furthermore, coaches differed greatly in their rationale repertoires—posing further challenges to interpretation. Coach rationales per game ranged from 16 to 42 (see Table 5).^6^ Such complexity and inscrutability poses serious challenges for interpreting rival behavior.

Our conceptual framework takes initial steps toward developing a theory for interpreting competitive actions. It reveals the key types and target domains of primary rationales. The three types of primary reasons—rivalrous, organizational, and social—indicate what managers *prioritize* with a particular action; that is, dealing with rivals’ actions, making organizational adjustments, or managing interpersonal issues. That specifies the reasons for an action. The framework also sheds light on the target domains or *scope* of each rationale: immediate issues versus long-term objectives, a single action versus strategy, and a direct opponent versus other stakeholder. Thus, our findings extend competitive dynamics research by getting behind “observable indicators to discover the perceptions and motivations that give rise to observable market actions” (Chen & Miller, 2012), in the process provoking a reevaluation of the AMC framework.

### Implications for the AMC Model

The AMC model has served as a central pillar for studying competitive action and response (e.g., Chen, 1996; Chen et al., 2007; Chen & Miller, 1994, 2012; Tsai, Su, & Chen, 2011). Our findings have implications for each of the model’s three components: awareness, motivation, and capability. First, our research suggests that the study of rationales can expand and deepen our characterization of competitor *awareness*, revealing hitherto unrecognized challenges to interpreting rival actions. Second, primary rationales explain *motivations* to act or forbear, reflecting longer term aims, parties beyond rivals, and broader strategic goals. Third, whereas prior research emphasized how capabilities drive competitive actions, we also find the reverse—that tactical actions *build* capabilities—for example by preserving and orchestrating resources to gain advantage in *future* periods. We discuss these findings below.

#### Awareness

Prior research has focused on factors influencing how managers identify, monitor, and perceive rivals and how they come to be aware of their actions (Chen & Miller, 2012). However, one can be aware of rivals and their actions, while not comprehending their rationales. Thus, understanding competitive rationales is essential for full awareness. Without it, actions remain opaque to interpretation. This can be challenging as our findings have shown that rationales are collectively complex and potentially inscrutable.

First, they differ across our types and target domains. They can be not only rivalrous but organizational and social, involving internal, relational, and strategic purposes difficult to detect or understand. They may address situations beyond a current attack, opponent, or short-term outcome to pursue undetectable purposes, such as preserving options, managing relationships with other parties, and concealing strategies. The resulting inscrutability is especially challenging as many rationales are *purposely* obscure, deceptive, and surprising (Guidice, Alder, & Phelan, 2009; Hendricks & McAfee, 2006). A second challenge to awareness is that rationale diversity—the range of rationale types—is far richer and broader than action diversity because multiple rationales can drive a single action.

Research on action-response dyads finds that awareness is influenced by an action’s observable characteristics—radicality, irreversibility, implementation requirements, and noteworthiness—all of which determine the likelihood and speed of response (Chen, Smith, & Grimm, 1992; Smith, Grimm, Chen, & Gannon, 1989). Our study recasts that externally imputed portrait of awareness, the limits of which surface when complex and inscrutable rationales hobble effective response. Similar limitations also apply to research on competitive *repertoires*, sets of competitive actions carried out by firms over a given period (Connelly et al., 2017; Ferrier et al., 1999; Miller & Chen, 1994). Simple repertoires with few types of actions are said to hurt performance (Miller & Chen, 1996). However, we find that identical actions can be motivated by many different rationales, making even simple repertoires potentially inscrutable and effective.

These findings enrich our appreciation of the potential subtlety, complexity, and challenges of competitive engagement in business. Indeed, if tactical rationales are so diverse, deceptive, temporarily nuanced, and broadly contextualized in basketball, that may also hold in business situations. There, managers with different priorities from different departments make decisions in a context of multiple rivals and stakeholders on a range of rival-directed, organizational, and social issues. Under such conditions, interpreting and explaining rivals’ rationales could be far more challenging than merely noticing their actions. Thus, our study suggests other aspects of awareness for scholars to consider in the AMC model: (1) To what extent can competitors distinguish rivalrous from social and organizational rationales? (2) How can managers assess rationale scope: for example, immediate situation versus longer term strategic goals and external parties? (3) How can managers recognize deceptive and misleading rationales?

#### Motivation

Competitive rationales disclose a multitude of motivations to act and respond or forbear. In prior studies, the *motivation* component of AMC concerned economic incentives and psychological motivations to act or respond in a competitive situation (e.g., Kilduff et al., 2010; Kilduff, Galinsky, Gallo, & Reade, 2016; Livengood & Reger, 2010; To et al., 2018; Tsai et al., 2011; Withers, Ireland, Miller, Harrison, & Boss, 2018). However, coach rationales divulge social and organizational motivating reasons for both action and *inaction* (Alvarez, 2010; Davidson, 1963; O’Connor, 2013). Our discussion of awareness illustrated the inscrutability of such motivations.

This challenge is compounded as forbearance reflected a critical and largely invisible and temporally conditioned effect of motivation. *Forbearance* occurs when managers are aware of an action and capable of responding, but for strategic purposes are motivated to not respond immediately (Andrevski & Miller, 2020). Table 5 shows that 112 (67%) of forbearance rationales were long-term and strategic (to adjust strategy, to orchestrate resources, and to learn). In our study, coaches chose forbearance to create *later* advantage over rivals, such as to surprise and mislead them in the future, preserve an option for more opportune use, take time to learn from experience, or grow capability. Forbearances also pursued longer term strategic organizational benefits, such as preserving strategic consistency or concealing a new strategy from future rivals. Thus, “managers will often strategically forbear when they look at the broader context of an attack, considering it in the light of parties other than the attacker, longer-term past and future events, and more systemic aspects of strategic cohesiveness and adaptation” (Andrevski & Miller, 2020: 10). It is important, therefore, that future scholars using the AMC model consider forbearance as a core aspect of motivation.

Interestingly, Table 5 suggests that more successful coaches chose forbearance more frequently. For example, forbearances accounted for 30% of all rationales for Coach 1, who had won six championships (and five second places), and 37% for Coach 9, who won four championships (and two second places). For comparison, all other coaches combined had only won two titles and their forbearance use averaged 17%.^7^

It may be that more accomplished competitors see the strategic value of forbearing, perhaps because they contemplate longer time horizons, broader contexts, and parties beyond the direct rival. It is possible too that accomplished competitors are more confident and better able to refrain from acting impulsively under pressure. Such discipline and composure manifested in forbearance may be critical to effective timing, resource orchestration, and misdirection (Andrevski & Miller, 2020; Chen & Miller, 2015).

Firms engaging in multimarket competition are especially likely to forbear strategically to achieve economic benefits (Gimeno & Woo, 1996; Yu & Cannella, 2013). Perhaps, as suggested above, such mindful engagement is especially likely to extend time horizons and the number of considered stakeholders and broaden strategic contexts; such careful reflection may yield superior returns. Multimarket competition research considers fear of retaliation across multiple markets as the primary reason for mutual forbearance. Our study suggests that multimarket rivals also might forbear to build resources for future attacks, optimize resource use, avoid conflicts with other stakeholders, or mislead rivals. We invite future researchers to assess these notions.

#### Capability

The *capability* to act and respond is also qualified by our findings. First, as noted, ignorance of primary rationales can thwart effective response, thus perhaps ultimately degrading the relevance of the stock of capabilities. Second, whereas the focus of prior studies has been on a rival’s *current* stock of capital and knowledge resources (Chen, 1996; Connelly et al., 2017; Miller et al., 2019), our coaches paid more attention to dynamically managing *their own team’s* capabilities rather than assessing those of rivals. Indeed, tactical actions often were intended to nurture, organize, and develop resources even in the throes of an intense competition. Thus, whereas AMC treats rivals’ relative capabilities in a largely static manner, our findings suggest that capability can be a very dynamic aspect of rivalry that builds over time and considers interactions with multiple rivals both in the moment and in the future. Interestingly, whereas prior research focuses on capability to carry out actions, we find a reverse sequence; actions are initiated to develop capabilities. This finding enriches competitive dynamics research that has not “adequately embraced time-based actions that focus on building future competitiveness” (Chen, Michel, & Lin, 2021: 15). Thus, tactics targeting longer term capabilities may play a subtle strategic role in competition.

In short, our research into rationales reveals a set of new opportunities for building and extending the AMC model in terms of neglected challenges to awareness, strategic aspects of motivation, and a more dynamic consideration of capabilities.

### Other Opportunities for Future Research

Competitive dynamics scholars often view competitive behavior from a rational, game theoretic perspective (Chen & MacMillan, 1992). However, as we have shown, the *human element* appears to be critical (see Kilduff, 2019; Kilduff et al., 2010). Our coaches attended to player morale and interaction, rival emotions, and the dignity of players and rivals. In inter-*firm* competition where reputation and firm image may matter greatly, similar considerations warrant further examination. Our coaches also considered multiple stakeholders of competitive engagement beyond direct rivals. In business, these would include customers, suppliers, and the public. Their roles in tactical competition remain to be explored (Chen & Miller, 2015).

*Action sequences* constitute an uninterrupted series of competitive actions (Ferrier, 2001; Rindova, Ferrier, & Wiltbank, 2010). Owing to rationale inscrutability, what rivals observe as a predictable sequence could instead be driven by diverse and unpredictable rationales. Moreover, coaches were careful to use the right players at the right times, to accelerate and slow down the pace of play as opportunities and threats evolved, and to attack and retreat when the time was right. This sequencing, pacing, or timing of behavior has been accorded less attention in the literature on competitive dynamics, and yet it is certain to figure very prominently in the behavior of businesses and the managers and entrepreneurs who run them.

In competitive dynamics, *the role of deception* appears to be a critical one and well worth further exploration (Sharapov & MacAulay, 2020). Coaches initiated “fake” moves to confuse opponents or refrained from carrying out expected moves to conceal information or intent, set a trap, and surprise rivals. Indeed, previous work has examined the ethical and moral aspects of competitive bluffing (Guidice, Alder, & Phelan, 2009), while microeconomists have studied the role of offensive feints in patenting decisions to mislead rivals (Hendricks & McAfee, 2006; Langinier, 2005). Our study extends this domain by revealing a wide range of misleading actions and forbearances that can make competitive *repertoires* inscrutable to rivals. Further work in this domain may provide unique insights into the antecedents and consequences of providing intentionally misleading or incomplete information about competitive rationales (McGrath et al., 1998; Sharapov & MacAulay, 2020).

#### Limitations

Certainly, our study has limitations. Its context is specific: There is only one decision-maker, the coach, and decisions take place in rapid sequence during a zero-sum game with clear rules. This is quite different from businesses where there are often multiple decision makers, with managers lower in the hierarchy making many tactical decisions, likely with fewer rules or temporal constraints than in basketball. In addition, although our coaches’ rationales did sometimes consider the morale of an opposing team, it is unclear how much such contemplation figures in competition among firms. Finally, the games we studied took place early in the season. It is uncertain whether the longer term strategic orientation of tactical rationales would change later on as championships became closer.

Moreover, we selected a competitive context in which rivals rely exclusively on tactical actions. Although this enabled us to reveal strategic rationales for tactical actions, it limited our ability to examine links between tactics and other major strategic actions. Also, basketball coaches make quick decisions with limited action possibilities and timeframes, whereas business managers often have time to analyze data, generate alternatives, and execute actions. The less hectic pace may allow managers to be more strategic. Thus, as noted, we might expect an even richer set of rationales for tactical actions in a business context. Finally, it would have been nice to compare coaches and their repertoires for effectiveness, but this was limited by our small sample.

## Conclusion

The field of competitive dynamics has been criticized as being undertheorized with too much emphasis on simple rational models and too little direct evidence of the complex human motivations underlying actual competition (Andrevski & Miller, 2020; Chen & Miller, 2012, 2015; Kilduff et al., 2010). Our investigation of tactical rationales reveals vibrant and multifaceted competitive rationales underlying observable rivalry. These rationales often reflect farsighted and subtle maneuvers, stratagems, and ploys driving tactical behavior. Clearly, studying observable tactics alone would reveal too little about the complex, sometimes ingenious, thinking behind them.

Our study broadens the foundations of competitive dynamics research, revealing that a single tactical action may be driven by a wide variety of competitive, organizational, and social rationales, many extending beyond a current exchange in time and parties implicated, and encompassing systemic and strategic considerations. Rationales revealed behavior that was purposefully deceptive, concealed, and deliberately withheld to surprise or mislead. Finally, rationale complexity and inscrutability could vary substantially across rivals, further giving rise to difficulties of interpretation and response, enhancing the competitive potential of tactical actions, and thereby importantly conditioning the classic AMC model to enhance the interpretability of tactical actions. In short, our findings suggest that the field can benefit from a more encompassing and nuanced portrayal of competitive interaction.

## Notes

1. Coaches’ statements sometimes involved multiple rationales. The statistics above are based on the coders’ first mentioned rationale for the statement. The index was much higher (0.86) when we counted any match between the coders’ rationales for each statement.

2. Eight rationales could be classified in another cell. For example, rationales such as “to surprise” or “to mislead” were mainly focused on tricking rivals for gaining short-term benefits. There were instances, however, in which the intended benefits had prolonged effects in the next quarters of the basketball game. Similarly, rationales in Cells III, VI, and IX primarily deal with external parties beyond current competitor, but they could also involve longer term strategic outcomes (e.g., to preserve rival dignity or to mislead future rivals).

3. A team includes coaches and players.

4. The identifiers AR (action rationale) and FR (forbearance rationale) correspond to the ID shown in Table 3, whereas the identifier CG (Coach #, Game #) in the text corresponds to the unique ID of a coach statement in our dataset not included in Table 3.

5. For example, coaches made a player substitution with a primary purpose to preserve rival dignity (AR 40; FR40), sanction a player (FR39), motivate a team (AR35; AR35), increase player confidence (AR36; FR36), create rival weakness or neutralize advantage (AR34; AR29), manage resource constraints (AR13; FR14; FR15), preserve and optimize resources (AR10; AR11), improve strategy execution (AR8), boost energy (AR6), or provide learning opportunities (AR3; FR3, AR4; FR4).

6. Whereas Coach 7 expressed 32.5 rationales per game, ranging from 23 to 42, Coach 4 averaged a similar number of rationales per game, 30.75, but with a much narrower range of 28 to 32 (see Table 5).

7. Although our study was not designed to confirm such findings statistically, out of curiosity we ran a mixed-effect logit model to tentatively assess whether these findings hold taking into account all observations and controlling for coach and team attributes (coach fixed effect, game effect, period of the game, time to interview, past game score, available players in the game, winning confidence, head coach experience). The results showed that coaches with more titles had 42% higher odds of forbearance (odds ratio = 1.42, *p* < 0.02), whereas head coach experience was unrelated to forbearance likelihood (these results are available on request).
